# Head Lice Infestation in Pre-High School Girls, Lak Hok Suburban Area, Pathum Thani Province, in Central Thailand

**DOI:** 10.1155/2023/8420859

**Published:** 2023-01-24

**Authors:** Sirima Kitvatanachai, Kanyanan Kritsiriwutthinan, Aree Taylor, Pochong Rhongbutsri

**Affiliations:** ^1^Faculty of Medical Technology, Rangsit University, Pathum Thani 12000, Thailand; ^2^Department of Preclinical Science, Faculty of Medicine, Thammasat University, Pathum Thani 12120, Thailand

## Abstract

This is a cross-sectional descriptive survey to determine the prevalence of lice infestation in a pre-high school, Lak Hok subdistrict, Pathum Thani Province, in central part of Thailand. The knowledge, attitudes, and practices of parents/guardians toward head lice in female children during February–April 2020 were evaluated. A total of 83 out of 111 parents (74.8%) agreed to complete consent forms and questionnaires. The prevalence of pediculosis found in this study was 68.7%. The infestation was found in the primary school level (87.5%), which was significantly higher than pre-primary school (29.6%; *p* < 0.05). Itching scalp was revealed in 93.0% of pediculosis. The results showed that occupation, religion, education, and income showed no significant difference between lice infested and non-infested students (*p* > 0.05). There was no significant association between sex, occupation, religion, education, income, status of parents, and family size among lice infested and non-infested students (*p* > 0.05). The parents/guardians showed the middle level of knowledge concerning with pediculosis capitis (66.8%). The lack of knowledge leading, first, to the belief that pediculosis does not need any treatment (89.2%), followed by boys getting head lice more frequently than girls (85.5%), and the belief that sharing infested combs, brushes, or hair ribbons does not result in lice transmission (79.9%). More than 75% of the parents/guardians had experienced infestation of head lice and showed themselves willing and able to diagnose and treat their family. However, 50.6% of them did not feel shame when their children were infested with head lice. Children washing their hair by themselves were found to be a significant factor in infestation (88.5%). High rate of pediculosis in this suburban school needs more intensive care by parents/guardians and teachers. Furthermore, improvement strategies to prevent and control lice in the school need to be more specifically planned and scheduled by teachers and health administrators.

## 1. Introduction

Pediculosis capitis remains a worldwide public health problem in developing and developed countries [1–3]. Hundreds of millions of people have been infested worldwide, ranging from 0% to 78.6% in different countries and areas [2, 3]. There is an estimated 19% global prevalence of head lice infestation among schoolchildren [2]. Transmission of head lice occurs mainly by direct close contact, or indirect transmission via hats, sharing contaminated combs, pillowcases, and clothes [4, 5]. People with infestation of head lice report itching (36% of cases) [6] due to the irritative effects of lice saliva, which in turn produces various signs, such as pruritus and eczematous changes [6, 7]. The head lice are potential vectors for *Rickettsia prowazekii*, *Bartonella quintana*, *Borrelia recurrentis* [8–11], and *Acinetobacter* spp. [8, 12]. Although 14.2% of the cases are symptomless [13], infestation also interferes with daily life, especially in school aged children, causing psychological stress in children and parents, which leads to frustration among parents [14, 15].

Head lice seem to be common in children between 3 and 12 years [16, 17], in girls more than in boys [18], and people of various social and economic status [17]. The prevalence of head lice infestation in Thailand varies from 15.1 to 86.1%; 86.1% in Ratchaburi school girls [19], 23.32% overall in schools, Eastern area of Bangkok, and 47.12% in girls [14], 15.1% overall in schools, Northern area, and no infection in boys [20]; and 37% overall in Ubon Ratchathani and no infection in boys [21], and 37.9% overall in rural Thailand and 50.1% in girls [22].

The policy of providing health services to communities led us to analyze community health problems by surveying lice infection in schools around Rangsit University. In 2017, 247 students in primary school, Lak Hok subdistrict, were checked for lice infestations. There was a 74.6% (68/89) rate of infestation in girls and 1.1% (1/89) in boys, with the prevalence of lice infestation in both sexes being 38.8% (69/178). We provided shampoo and fine combs for all infestations. Later, in 2018, a campaign to stress the elimination of lice infestations was conducted by a survey of pediculosis, focusing on girls and providing knowledge to all teachers, parents, and students in order to protect them from lice infestations. Lice infestation was found to have increased to 89.2% (83/93), and, surprisingly, in some classrooms, there was a 100% infestation rate (Unpublished data from faculty of Medical technology, Rangsit University, 2018). This encouraged us to find out key ideas in knowledge, attitudes, and practices of parents/guardians on how to reduce the morbidity of pediculosis capitis, since previously our campaign only focused on students' practices. The results of this study may be useful in making strategies for the next intervention to control pediculosis in suburban areas, which will concern health administration, teachers, and students' families. Therefore, our study also aims to follow up the prevalence of lice infestation and to determine the association of knowledge, attitudes, and practices of parents/guardians toward pediculosis in all girls in pre-high school in Lak Hok suburban area, Pathum Thani Province, Central Thailand.

## 2. Materials and Methods

### 2.1. Study Design and Study Participants

This was a cross-sectional study from February to April 2020 among pre-high schoolchildren (aged between 4 and 12 years): pre-primary school (aged 4–6 years) to primary school (aged 7–12 years) girls at a suburban school located in Lak Hok subdistrict of Pathum Thani Province in central part of Thailand, which is approximately 4–5 km away from Rangsit University. There were 111 girls in school at that time. The inclusion criteria were girls who were studying in this school, obtained agreement from parents/guardians to undergo investigation for lice infestation, and whose parents/guardians filled out the questionnaires. The exclusion criteria were boys and girls who were absent from the school at that time or did not agree to participate.

This study was approved by the Human Ethical Review Committee of Rangsit University, Thailand (ethical clearance no. RSUERB 2020-008). Written informed consent was obtained from each child's parent or guardian, and permission to study was granted by the school's director.

### 2.2. Determination of Head Lice Infestation in Girls

Lice infestation in this study was observed for active infestation based on visual detection of the living adult, nymphal stages, or viable nits (nits that were found less than 0.6 cm away from the scalp and brown in color) under natural sunlight [23–27]. A positive case was considered as the detection of at least one living head louse or nymphal stage, or viable nits with adult or nymph [25]. If no nymphs or adults were seen, then the infestation was probably old and no longer active; this was assumed as negative [28].

### 2.3. Data Collection

A structured questionnaire was developed following [18, 29]. The questionnaire consisted of four categories of questions for parents/guardians to answer including: (1) socio-demographic characteristics of the respondents (7 questions), (2) knowledge of head lice and pediculosis (20 true or false questions), (3) attitudes of parents/guardians toward lice infestation (10 yes or no questions), and (4) practices of parents/guardians regarding head lice in female children (10 multiple choice questions).

These materials were checked by three specialists in parasitology and medical education technologists. The pre-test questionnaire was conducted in one school in the same area of Lak Hok subdistrict and adjusted for structure and reliability before being used. The parents/guardians were asked to participate in this study and signed a consent form. The questionnaires were passed to parents/guardians by teachers via their students. All class teachers were trained to investigate lice infestation. Lice infestation was diagnosed and recorded by a researcher team on the same day of questionnaire collection and followed up by class teachers. All lice infested students were provided with a fine comb, 30 ml of herbal shampoo (*Annona* leaves mixture), and instructions to protect themselves from lice infestation.

### 2.4. Data Analysis

Data entry and analysis were done by using the IBM SPSS software for Windows (version 21.0). Descriptive statistics were used to describe the prevalence of lice infestation in percentage. The results were presented as frequencies and percentages for qualitative variables.

(1) $$\begin{array}{c} \text{The prevalence of lice infestation }\left(\%\right)=\frac{\text{number of positive cases}}{\text{total no}.\text{of investigated sample}}\times 100. \end{array}$$

Chi-square test was used to determine the association of knowledge, attitudes, and practices of parents/guardians, and pediculosis in girls in pre-high school in Lak Hok suburban area. Variables with a *p*-value <0.05 were declared as statistically significant.

## 3. Results

### 3.1. The Prevalence of Head Lice Infestation in a School in Lak Hok Subdistrict

A total of 83 out of 111 girls (74.8%) obtained agreement from parents/guardians to participate in this study. The prevalence rate of pediculosis among 83 girls in pre-high school in Lak Hok subdistrict was 68.7% (57/83). The highest prevalence reported in primary school grade 5–6 was 95.5%, followed by primary school grade 1–4 (82.4%), and the lowest infestation in pre-primary school (29.6%), with statistically significant results (*p* < 0.05; Table 1). The itching symptom was reported in 53 out of 57 girls (93.0%).

### 3.2. Socio-Demographic Data of Parents/Guardians and the Prevalence of Head Lice Infestation in a School in Lak Hok Subdistrict (*n* = 83)

The distribution of the socio-demographic characteristics of parents/guardians including sex, occupation, religion, education, income, status of parents/guardians, and family size (Table 2). Lice infestation in pre-high schoolchildren in Lak Hok subdistrict was found at the highest rate in the parents/guardians group who were general workers (76.7%), income >16,000 baht/month (77.8%), living with father/mother and no clarification of parent's status (75–100%), and staying with family >5 persons (72.4%). However, none of them were statistically significant (*p* > 0.05; Table 2).

### 3.3. Knowledge of Head Lice Exhibited by Parents/Guardians of Schoolchildren in Lak Hok Subdistrict

Approximately 63.8% (53/83) of parents/guardians showed medium knowledge of head lice (50–79%), and only 15.7% of parents/guardians showed poor knowledge (less than 50%; Table 3). Parents/guardians were found to have highly deficient knowledge of lice infestation that can be resolved without treatment (89.2%), followed by boys getting head lice more frequently than girls (85.5%), sharing infested combs, brushes, or hair ribbons resulting in lice transmission (79.9%), close contact with lice infested people transmitting head lice (66.3%), lice infestation affecting sleep (65.1%), and the fact that if a louse falls off a person, it dies within 1–2 days (63.9%; Table 3).

### 3.4. Parental Attitudes Concerning Head Lice Infestation among Schoolchildren in Lak Hok Subdistrict

Most parents/guardians have seen head lice and nits (90.4% and 92.8%, respectively). They were able to take responsibility for both the detection and treatment of head lice (78.3% and 75.9%, respectively). Meanwhile, 50.6% experienced feeling shame and embarrassment when their children were infested with *Pediculus humanus capitis* (Table 4).

### 3.5. Parental Practices Concerning of Head Lice Prevention among Schoolchildren in Lak Hok Subdistrict

The highest lice infestation was shown in students who washed hair everyday (76.2%), washed hair by themselves (88.5%), showered 1–2 times a week (75.0%), always shared comb, clothes, or towel (71.4%), cleaned bedsheet/pillowcases one time a month (83.3%), exposed bedsheet to the sun one time a year or never (100%), and lived with father/mother/relatives who had lice infestation (100%; Table 5).

## 4. Discussion

There are no published reports on pediculosis in Pathum Thani Province. In 2017, Rangsit University recognized the importance of health services to communities, and we were assigned to analyze parasitic infections in schools around Rangsit University. We found that pediculosis was only a serious public health problem in a school with the prevalence of 38.8% (69/178), 74.6% (68/89) in females, and only 1.1% in males. A pack of 20 ml of anti-lice shampoo (0.5% permethrin w/w) and fine combs was provided for all cases of infestation. Later, in 2018, lice infestation was found to have increased to 89.2% (83/93) in female students. A campaign to eliminate lice infestation was conducted, focusing on girls and providing more anti-lice shampoos. The knowledge of pediculosis capitis was also presented to all teachers, parents, and students, so they could protect themselves from lice infestation. However, many parents/guardians (~50%) had no time to participate. This study, in a suburban area of Lak Hok, Pathum Thani Province, showed the prevalence of lice infestation had decreased from 89.2 to 68.7% in girls. This was a higher prevalence than previously reported: 47.12% in the eastern area of Bangkok [14], 15.1% in Northern Thailand [20], 37% in Ubon Ratchathani Province [21], and 50.1% in rural Thailand [22]. However, this was a lower prevalence than in primary schools near the Thai-Myanmar border in Ratchaburi Province, Thailand (86.1%) [19]. Pediculosis is more prevalent among girls [18, 19]. A surprising result was the long-term high prevalence in a suburban school. This needs to be seriously focused on by teachers, students, and health ministering to create a policy of eliminating lice infestation. Treatment of head lice involves insecticides and mechanical removal [30]. Treatment failure can be attributed to lice resistance to insecticides [31–34], too short an exposure time or insufficient dosage. Treatment must be repeated after 7 days to kill newly hatched nymphs [35]. Our previous surveys, in which we provided 20 ml of anti-lice shampoo, which included permethrin mixtures, might not have been enough to treat pediculosis and were not repeated.

Lice resistance to permethrin has been reported [36]. Thus, alternative herbal shampoos were given in a bottle (30 ml) for all pediculosis in this study. Unfortunately, we could not arrange time to follow up the results of the shampoo treatment. The rate showed significantly higher prevalence of pediculosis capitis in primary school (83.1%) than pre-primary school (29.6%; *p* < 0.05). More interesting, 95.5% of lice infestation was found in grade 5–6 students, which contrasts with Thanyavanich et al., who reported the highest prevalence in grade 1–3 and pre-primary school [19]. This might be due to pre-primary schoolchildren in this area live with and are intensively cared for by parents/teachers, and not many kids study in a room (10–20 kids). During primary school, there are much higher numbers of students per room (20–30 students), and at this level, students have more activities with their friends. Since most parents (~75%) earn lower income (16,000 baht/month), the parents finish work late and leave them with guardians. Lice infestation is of a continued high prevalence in this school, and primary schoolchildren may not realize how harmful this insect is, and at this age, personal hygiene was taken care of by students.

Due to the high rate of infestation, there were no significant differences between occupation, education, income, status, or family size of parents/guardians, and lice infestation (*p* > 0.05) in this study.

In this study, only 15.7% of respondents failed to answer less than 50% of questions correctly. However, the infestation was found to be the highest in the children of parents with the highest knowledge, even though it was not significant (*p* > 0.05). Thirty five percent of Australian parents reported a lack of knowledge on pediculosis, which is higher than shown in this study [37]. Moreover, the lack of knowledge also extended to health professionals [38, 39]. It was found that there was deficient knowledge regarding: (1) lice infestation can be resolved without treatment (89.2%), which is generally believed worldwide, and treatment or a visit to a health care center is not required [40], (2) boys get head lice more frequently than girls (85.5%), (3) sharing infested combs, brushes, or hair ribbons does not result in lice transmission (79.9%), (4) close contact with lice infested people is a way of head lice transmission (66.3%), (5) lice infestation does not affect sleep (65.1%), and (6) if a louse falls off a person, it dies within 1–2 days (63.9%). In attitudes concerning pediculosis, it is noticeable that 49.4% did not feel embarrassment when their children were infested with *P. humanus capitis.* In contrast, in developed economies, distress about being infested results in feelings of disgust, horror, dirtiness, frustration, and anxiety [41, 42]. This belief is a reason that the parents avoid seeking treatment.

In this current study, pediculosis was found at the highest prevalence in students who washed their hair by themselves (88.5%), which was significantly higher than other groups (*p* < 0.05). Even in students who washed hair daily, the high infestation was still reported if they did it incorrectly.

Our findings may be a message to further tailor interventions for students and families, as well as teachers. Arrangements and evaluations may be needed continuously for sustainability.

## 5. Conclusion

High prevalence of lice infestation (68.7%) was reported among primary school girls in Lak Hok suburban area, Pathum Thani Province, Central Thailand. The health education program in pediculosis is needed to target parents/guardians, students, as well as teachers, in order to increase knowledge and change some attitudes toward preventive practice. There is an urgent need for proper lice treatment and repeat treatment to eliminate lice infestation.

## 6. Limitations

Due to the COVID-19 epidemic in 2021, we could not follow up the prevalence on lice infestation after use of herb shampoo. This study included only one school from our previous survey that showed a high rate of infestation. Parents/guardians in this area had almost no time, because of routine work, to participate, and they did not acknowledge the importance of lice infestation.

## Figures and Tables

**Table 1:** The prevalence of head lice infestation and itching sign of infestation cases in schoolchildren in Lak Hok subdistrict.

Level of studies	Total		Lice infestation		No infestation		*p*-Value
	No.	%	No.	%	No.	%	
Pre-primary school (aaged 4–6 years)	27	32.5	8	29.6	19	70.4	0.001^∗^
Primary school							
Grade 1–4 (aged 7–10 years)	34	41.0	28	82.4	6	17.6	
							
Grade 5–6 (aged 11–12 years)	22	26.5	21	95.5	1	4.5	
Total	83	100.0	57	68.7	26	31.3	

∗Chi-square test showed statistically significant.

**Table 2:** General socio-demographic data of parents/guardians and the prevalence of head lice infestation in schoolchildren in Lak Hok subdistrict.

Characteristics of parents/guardians	Total (*n* = 83)		Lice infestation (*n* = 57)		No infestation (*n* = 26)		*p*-Value
	No.	%	No.	%	No.	%	
Sex							
Male	8	9.6					
Female	75	90.4					
Occupation							
No work	9	10.8	5	55.6	4	44.4	0.787
Trade/business	21	25.3	15	71.4	6	28.6	
General worker	30	37.3	23	76.7	7	23.3	
Government officer	3	3.6	2	66.7	1	33.3	
Private employees	2	2.4	1	50.0	1	50.0	
Housewife	13	15.7	8	61.5	5	38.5	
Other	5	6.0	3	60.0	2	40.0	
Religion							
Buddhism	82	98.8	56	68.3	26	31.7	
Christianity	1	1.2	1	100.0	0	0	
Education							
Illiterate	1	1.2	1	100.0	0	0	0.232
Primary school to secondary school	69	83.1	45	65.2	24	34.8	
Bachelor degree and above	8	9.6	7	87.5	1	12.5	
No answer	5	6.0	4	80.0	1	20.0	
Income (baht)/month							
<9000	37	44.6	26	70.1	11	30.0	0.870
9,001–16,000	25	30.1	17	68.0	8	32.0	
>16,000	9	10.8	7	77.8	2	22.2	
No answer	12	14.5	7	58.3	5	41.7	
Status of parents/guardians							
Father/mother	36	43.4	23	63.9	13	36.1	0.578
Relative	32	38.6	22	68.8	10	31.2	
Father/mother and relative	12	14.5	9	75.0	3	25.0	
No answer	3	3.6	3	100.0	0	0	
Family size							
≤5	54	65.1	36	66.7	18	33.3	0.139
>5	29	34.9	21	72.4	8	27.6	

**Table 3:** Knowledge of head lice exhibited by parents/guardians in schoolchildren in Lak Hok subdistrict.

Questions (correct answer)	No. of answers	
	True (%)	False (%)
1. Head lice are parasitic insects (true)	77 (92.8)	6 (7.2)
2. Head lice can live up to 1 year on a person's head (false)	18 (21.7)	65 (78.3)
3. If a louse falls off a person, it dies within 1–2 days (true)	30 (36.1)	53 (63.9)
4. Head lice can fly from person to person (false)	41 (49.4)	42 (50.6)
5. Head lice can be contracted from animals, such as cats and birds (false)	39 (47.0)	44 (53.0)
6. Boys get head lice more frequently than girls (false)	71 (85.5)	12 (14.5)
7. Head lice infestations occur only in developing countries (false)	45 (54.2)	38 (45.8)
8. Close contact with lice infested person is a way of head lice transmission (true)	28 (33.7)	55 (66.3)
9. High risk of lice infestation when live in crowded area (true)	71 (85.5)	12 (14.5)
10. Sharing infested combs, brushes, or hair ribbons does not result in transmission (false)	66 (79.9)	17 (20.5)
11. Pediculosis is a transmitted disease (true)	49 (59.0)	34 (41.0)
12. Itching is always present in lice infestation (true)	80 (96.4)	3 (3.6)
13. Most children with head lice frequently scratch their heads, which may lead to skin sores and skin infections (true)	71 (85.5)	12 (14.5)
14. Lice infestation does not affect sleep (false)	54 (65.1)	29 (34.9)
15. Lice infestation can be resolved without treatment (false)	74 (89.2)	9 (10.8)
16. All available products kill all the lice and their eggs (false)	37 (44.6)	46 (55.4)
17. Parents/guardians should ask a healthcare provider before using lice-killing products (true)	66 (79.5)	17 (20.5)
18. Use extra amounts of lice-killing medication to get more efficacy of treatment (false)	33 (39.8)	50 (60.2)
19. During infestation, delouse by using warm water to wash all clothing and bedding (true)	35 (42.2)	48 (57.8)
20. Wash hair at least two times a week to protect lice infestation (true)	65 (78.3)	18 (21.7)
Correct answers		No. infestation (%)
Less than 10 questions (<50%) correct	13 (15.7)	9/13 (69.2)
10–15 questions (50–79%) correct	53 (63.8)	33/53 (62.3)
16–20 questions (80–100%) correct	17 (20.5)	15/17 (88.2)
*χ* ^2^ = 4.038. df = 2. *p* = 0.133		

**Table 4:** Parental attitudes concerning head lice prevention in schoolchildren in Lak Hok subdistrict.

Questions	Yes	%
1. Have you seen head lice?	75	90.4
2. Have you seen head lice eggs (nits)?	77	92.8
3. Do you feel adequately able to differentiate between head lice and other insects?	68	81.9
4. Do you feel adequately able to differentiate between head lice eggs and dandruff?	64	77.1
5. Do you feel adequately able to check your family for head lice?	65	78.3
6. Do you feel adequately able to treat your family for head lice?	63	75.9
7. Do you feel shame when your child is infested with head lice?	42	50.6

**Table 5:** Parental practices concerning head lice infestation and percentage of their children infested in a school in Lak Hok subdistrict.

Parental practices	Total		Lice infestation		No infestation		*p*-Value
	No.	%	No.	%	No.	%	
1. Washing child's hair per week							
Everyday	21	25.3	16	76.2	5	23.8	0.686
1–2 times	23	27.7	15	65.2	8	34.8	
3–4 times	39	47.0	26	66.7	13	33.3	
2. Who washes child's hair							
Themselves	52	62.7	46	88.5	6	11.5	0.001^∗^
Father/mother	20	24.1	6	30.0	14	70.0	
Relative	10	12.0	4	40.0	6	60.0	
3. Child shower per week							
Everyday	79	95.2	54	68.4	25	31.6	0.629
1–2 times	4	4.8	3	75.0	1	25.0	
4. Sharing comb, clothes, or towel							
No	45	54.2	31	68.9	14	31.1	0.575
Yes	38	45.8	26	68.4	12	31.6	
5. Cleaning bedsheet/pillowcases							
One time a week	65	78.3	42	64.6	23	35.4	0.107
One time a month	18	21.7	15	83.3	3	16.7	
6. Exposing bedsheet to the sun							
Never	2	2.4	2	100.0	0	0.0	0.568
Everyday	19	22.9	13	68.4	6	31.6	
One time a week	48	57.8	32	66.7	16	33.3	
One time a month	12	14.5	9	75.0	3	25.0	
One time a year	1	1.2	1	100.0	0	0.0	
7. Lice infestation in family							
None	53	63.9	32	60.4	21	39.6	0.192
Brother/sister	22	26.5	17	77.3	5	22.7	
Father/mother	1	1.2	1	100.0	0	0.0	
Relative	3	3.6	3	100.0	0	0.0	

∗Chi-square test showed statistically significant.

## Data Availability

The data used to support the findings of this study are included within the article.
